# Candidemia before and after the COVID-19 pandemic: An analysis of risk factors and outcomes in patients with candidemia

**DOI:** 10.1017/ash.2022.204

**Published:** 2022-05-16

**Authors:** Nora Colburn, Courtney Nichols, Mark Lustberg, Shandra Day, Michael Haden, Christina Liscynesky

## Abstract

**Background:** An increase in candidemia has been observed throughout the world since the start of the COVID-19 pandemic. Patients with COVID-19 may have different risk factors, clinical presentations, and outcomes compared to patients without COVID-19. **Methods:** We conducted a retrospective chart review of all inpatients with candidemia at a large, academic medical center from April 30, 2019, to February 19, 2021. The first case of COVID-19 was detected at our institution March 2020 and patients were sorted into pre– versus post–COVID-19 pandemic groups. Data regarding clinical characteristics, risk factors, and outcomes were collected. The rate of candidemia per 10,000 patient days was calculated from January 2013 through February 2021. **Results:** In total, 202 patients were identified with candidemia: 92 cases were identified before the pandemic and 110 cases were identified after the pandemic began. Moreover, 33 (16.3%) patients were diagnosed with COVID-19 during the admission and 169 (83.7%) did not have COVID-19. Patients with COVID-19 were significantly more likely to be older (median, 64.5 vs 54.8 years; *P* = .0006) and to have a higher body mass index (32.8 vs 29.1; *P* = .03) than patients without COVID-19. Patients with COVID-19 were less likely have some of the traditional risk factors (eg, abdominal surgery, total parenteral nutrition, history of injecting drugs) for candidemia compared to patients without COVID-19. Patients with COVID-19 were significantly more likely to require ICU care (97.0% vs 67.5%; *P* < .001) and to require mechanical ventilation (90.9% vs 53.9%; *P* < .001), and they had higher mortality at 30 days (66.7% vs 31.4%; *P* < .001). A multivariate logistic regression model showed that COVID-19 (OR, 2.53; 95% CI, 1.09–5.90) and higher age (OR 1.45, 95% CI, 1.11–1.91) were significant predictors of 30 day mortality. Using a Poisson regression model, the incidence rate ratio for candidemia per month after the start of the COVID-19 pandemic was 2.09 (95% CI, 1.85–2.36; *P* < .0001) compared to the years prior. **Conclusions:** Rates of candidemia significantly increased after the start of the COVID-19 pandemic. Patients with candidemia in the post–COVID-19 era tend to have nontraditional risk factors, to be more critically ill, and to have increased mortality compared to patients in the pre–COVID-19 era. COVID-19 and higher age were independent predictors of mortality. More studies are needed to further define risk factors for candidemia in patients with COVID-19.

**Funding:** None

**Disclosures:** None

